# Four‐year experience with an in‐house treatment management platform to streamline departmental operations in radiation oncology

**DOI:** 10.1002/acm2.70515

**Published:** 2026-02-24

**Authors:** Grace N. Chang, Yi Rong, Courtney Buckey, Matthew J. Susen, Pedro R. Lara, Pamela R. Lemish, Gregory S. Boutis, Quan Chen, Edward L. Clouser

**Affiliations:** ^1^ Department of Radiation Oncology Mayo Clinic Arizona Phoenix Arizona USA

**Keywords:** programming, quality management, radiotherapy data management, scheduling, task tracking, treatment planning whiteboard, workflow optimization

## Abstract

**Purpose:**

To develop and implement an in‑house treatment‑management platform to improve communication, data accessibility, and task tracking across a radiation oncology department.

**Methods:**

We developed Capsule, a custom software platform that centralizes patient data from the ARIA oncology information system (Varian Medical Systems, Palo Alto, United States), and integrates it with data from Outlook (Microsoft, Redmond, United States), spreadsheets, and manual inputs. Built using C#, SQL, and the .NET framework, it features a graphical interface in XAML with twelve modular tools to support workflows across a large multi‐modality department. A formal validation, feedback, and maintenance cycle was established. Its impact on workflow efficiency was evaluated using average CarePath task duration before and after implementation, supplemented by user interviews and surveys.

**Results:**

The Department Schedule, Treatment Planning and On‐Treatment Whiteboard, Spreadsheet, and various workload tracking modules emerged as the most frequently used among dosimetrists surveyed.The Whiteboard module consolidates planning metadata for streamlined workflows. Workload and scheduling modules help distribute tasks fairly. The Department Schedule module centralizes daily role and contact information. The Spreadsheet module allows dosimetrists to identify a reference for unique plans and provides secure access to large exportable tabulated patient data and clinical trends. Other modules support proton down‐time replanning and special procedure physics assignment. One year after Capsule's implementation, most tasks in the external beam radiation therapy workflow experienced a reduction in average duration.

**Discussion:**

Capsule streamlines workflows by integrating multiple tools into a single platform, supporting urgent scenarios, and improving task hand‐off. Its modular design allows customization to local workflows, but implementation must be guided by institutional oversight and formal quality management to ensure data security and compliance.

**Conclusion:**

Capsule can provide a solution for improved workflow efficiency, communication, and interdisciplinary coordination. By democratizing access to routine and retrospective data, it supports treatment planning, scheduling, and quality improvement. Ongoing updates are essential as clinical practices evolve.

## 1 INTRODUCTION

Efficient management of patient data across disparate systems remains a persistent challenge in radiation oncology. Relevant information typically resides in three primary systems: the Electronic Medical/Health Record (EMR/EHR), the Oncology Information System (OIS), and the Picture Archiving and Communication System (PACS). Quality care depends on the comprehensive transfer of data between these systems and to individuals.^1^ Despite efforts to consolidate charts in the EMR, PACS, or OIS, an abundance of patient‐specific information remains scattered among care teams and communicated informally. Verbal exchanges, sticky notes, emails and online messaging frequently go undocumented, thereby presenting opportunities for error or confusion.^2^

Beyond improving documentation of ad‐hoc information, communication efficiency can be enhanced by minimizing repetitive information requests. Centralized and democratized data can achieve that, allowing clinicians to focus on high‐priority and non‐automatable tasks.^3^, ^4^ As resources allow, documentation should be made easily accessible, routine tasks automated, and spreadsheet‑based tools replaced with automatically updated formats to enhance reliability.^5^, ^6^

Although enhanced documentation and data centralization can streamline routine communication, it does not address the issue of proactive communication needed for task hand‐off. Vendors typically provide systems to manage this, such as the CarePath functionality within the ARIA oncology information system (Varian Medical Systems, Palo Alto, United States).^7^ Other vendors provide similar functionalities within their Record and Verify products including workflow management tools in RayCare (RaySearch Laboratories, Stockholm, Sweden), Quality Check Lists from MOSAIQ (Elekta, Stockholm, Sweden) and a suite of SmartClinic workflows in Elekta ONE (Elekta, Stockholm, Sweden). In our Record and Verify System ARIA, a CarePath is a predefined sequence of tasks, decision points, and documentation steps that guide the multidisciplinary care team through the radiation therapy process from simulation to treatment planning, delivery, and follow‐up. These systems play a significant role in task hand‐off but are not comprehensive and store valuable data in formats that are challenging or inefficient to access.^8^, ^9^, ^10^, ^11^, ^12^

Several groups have acknowledged limitations of vendor systems and implemented whiteboards as in‐house solutions to reduce task time and last‐minute plans.^9^, ^10^, ^12^, ^13^ The proposed whiteboards serve as a comprehensive overview of a group of patients' progress throughout the radiation therapy workflow, rather than one patient at a time. Commercial versions, such as Elekta SmartBoards and RayCare Task‐based whiteboards, have begun to adapt to this concept but remain costly and dependent on proprietary integrations. Published in‑house solutions are typically more flexible but lack key metadata and fail to leverage available data to provide solutions for specific but common urgent tasks such as replans, machine down‐time, time sensitive procedures, workload review, and more.^9^, ^10^, ^12^, ^14^, ^15^ Herein, we present an in‐house software locally referred to as “Capsule”, developed to augment existing CarePaths and address gaps in data management. In addition to a whiteboard, it enables democratization of scheduling and clinical‑assignment data and facilitates the integration of non‑standardized clinical information to support workflows for both urgent and routine tasks.

## 2 METHODS AND MATERIALS

Capsule is a custom‐built software platform designed to support a multidisciplinary radiation oncology team of more than 200 staff members and approximately 2000 patients annually. Extending the functionality of whiteboard tools, Capsule includes 12 modular components designed to improve communication, task coordination, and workflow transparency. While module design is tailored to institutional needs, the underlying framework emphasizes translatable safety, quality, and data management.

### 2.1 Software architecture

Capsule is implemented with .NET software development framework using the C# programming language. Once retrieved from various input sources, the data is stored in a structured query language (SQL) database hosted on an institutional server, which is backed up regularly. The graphical user interface (GUI) was built using the Windows Presentation Foundation framework and Extensible Application Markup Language (XAML). Capsule's code is held in the department's code repository (Azure Development Ops Git).

### 2.2 Data retrieval and storage

**FIGURE 1:**
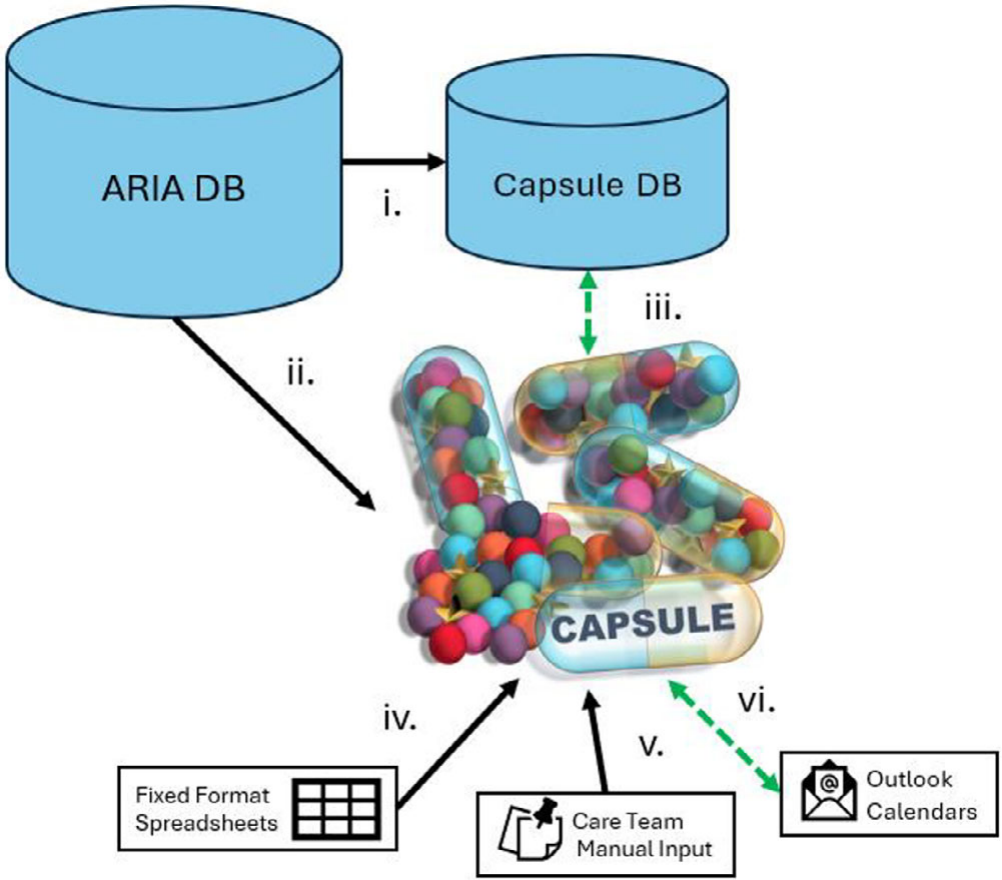
Dataflow for Capsule. Diagram displaying the flow of data into and out of Capsule. Dashed green indicates bidirectional reading and writing, black indicates unidirectional data transfer. (i) ARIA database (DB) to Capsule DB: Transferred data includes staff assigned to CarePath tasks, task progression, changes in appointments, and treatment plan data. (ii) ARIA DB to Capsule User Interface (UI): The Capsule UI can display ARIA DB query results, such as the number of events per day (treatments, special procedures, etc.). (iii) Capsule DB to Capsule UI: The Capsule DB reads and writes from the UI (manual input in Dosimetry Notes, special procedure scheduling). (iv) Spreadsheet to Capsule UI: Capsule's UI can read in a file selected by the user and convert the data into DB items for use in other Capsule modules, such as staff scheduling information. (v) Care team to Capsule UI: The Care team interacts with the Capsule UI to perform special procedure assignments and enter Dosimetry Notes. (vi) Outlook to Capsule UI: Outlook Calendars are read for department groups that manage their schedules directly in Outlook, for use in Capsule's aggregated department schedule. This includes items such as work assignments, PTO and meeting attendance, as well as work location for hybrid employees (on campus vs. remote). Outlook calendar invites are sent via the UI.

A summary of data sources, their flow and use is available in Figure 1 and in more detail in the Supporting Information (Table S1). Capsule retrieves data from the ARIA Record and Verify system via a dedicated Windows service running on a hospital‐hosted server. Using Microsoft's Entity Framework and LINQ, ARIA's Structured Query Language (SQL) data is translated into C# (Microsoft, Redmond, United States). Activity serial numbers associated with CarePath tasks are appended to a dictionary maintained in Capsule's database, along with associated status, personnel who have claimed the CarePath task, and timestamps. New patients are added to the Capsule database from their simulation appointment. The rest of the CarePath is added by therapists at the time of simulation and the flow of the patient through Capsule's various systems begins. Capsule's database interacts with ARIA in 2‐min intervals, updating when a task is marked as completed and the CarePath advanced.

**FIGURE 2:**
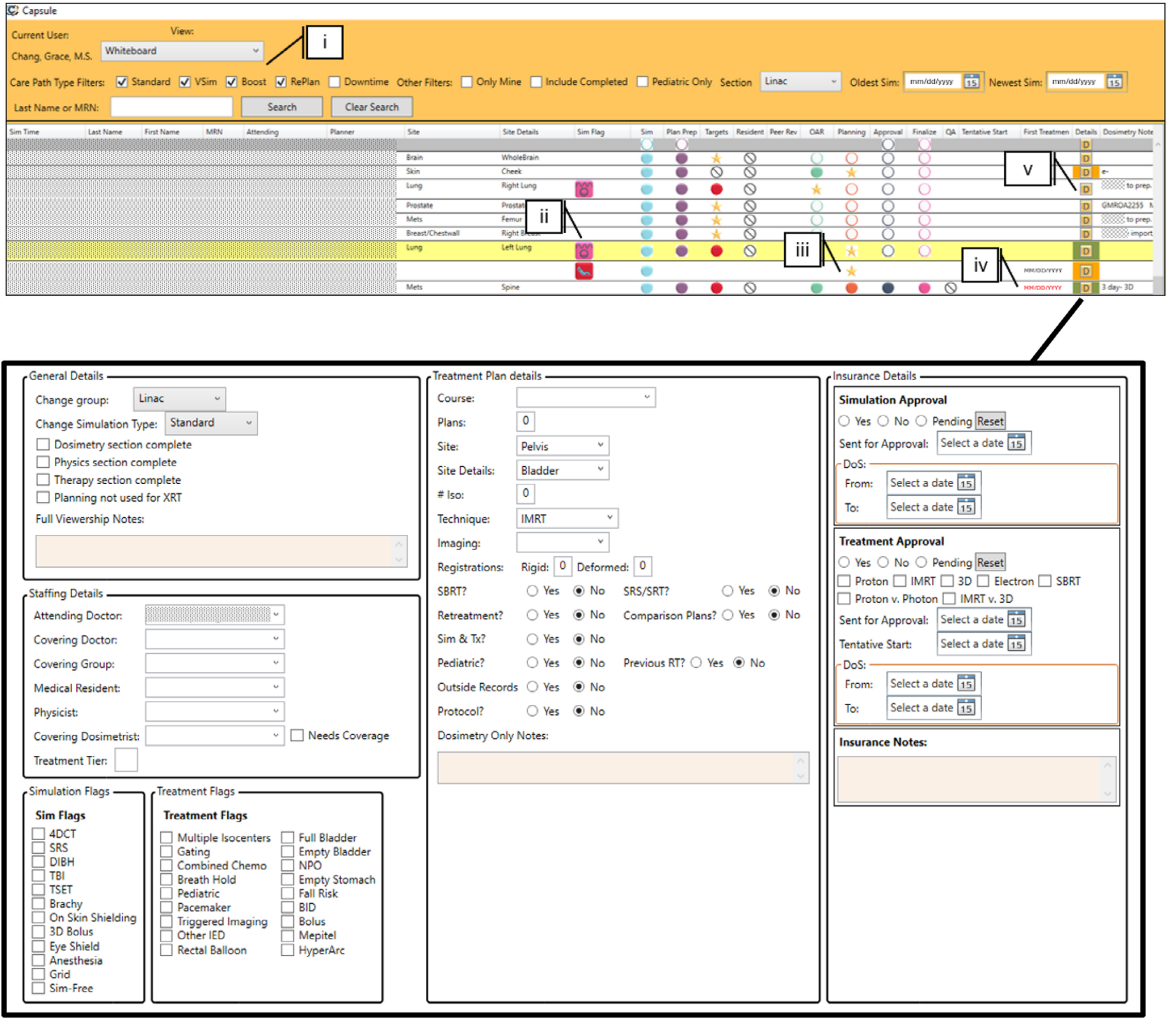
Capsule design choices featuring the Whiteboard module and Dosimetry Notes. Color and design choices are displayed in the Whiteboard module. (i) Filters are available in the top bar to sort based on type of CarePath, Linac or Proton, and sim date ranges. (ii) Additional metadata is available in iconographic formats and row color, for example, white corresponds to a standard simulation, whereas yellow indicates a replan. (iii) Status of CarePath is indicated by a star icon, with tasks to be completed indicated by a hollow circle and cancelled tasks with a strike through. Hovering over the task circles will display date and time of availability. (iv) When a patient's start date is approaching, the “First Treatment” date will turn red. (v) Dosimetry notes houses information not available in the OIS or EMR, displayed in the bottom panel.

In addition to personnel and patient data pulled from ARIA, external data sources include Excel spreadsheets and Outlook calendars (Microsoft, Redmond, United States). Daily information specific to employee coverage of tasks or assignments includes “Physicist of the Day,” special procedure coverage, urgent chart triage, and on‐campus versus remote working for hybrid employees. To perform employee scheduling, Capsule parses and stores existing scheduling spreadsheets, then sends calendar invites to the appropriate personnel from their group's shared calendar. Both Excel and Outlook provide publicly available Dynamic Link Libraries (DLLs) that enable these scheduling and data‑parsing functions (Office.Interop.Outlook and Office.Interop.Excel). To document previously scattered communication, treatment planners enter supplemental patient information into Dosimetry Notes (Figure 2.v). Examples include insurance approval status and updates, outside record status, clinical protocol enrollment, disease‐specific planning indications beyond the ICD‐10 code (e.g., which standard operating procedure to use), or other treatment flags. Treatment flags serve a dual purpose, both during treatment planning and when performing retrospective database mining. Importantly, this data is not consolidated in a sufficiently accessible way in any other database system.

### 2.3 User interface

Capsule is composed of 11 clinical modules and one administrative module, which is only available to trained individuals with elevated user rights (Super Users) (Table 1). Upon opening the executable stored on a department network drive, the program performs an automatic login that references the users’ Windows credentials so that preferences are preserved across workstations. The Capsule database uses the same ActiveX user group as ARIA, to prevent non‐ARIA users from accessing Capsule and can only be used inside the hospital's firewall.

**TABLE 1:** Summary of Capsule functionality and the main user groups.

Module	User group	Purpose
Calendar	Therapy	Provide overview of daily, weekly, and annual patient load
Chart Rounds	Dosimetry Assistants	Assist in preparing materials for chart rounds and ensuring all new starts are discussed
Case Totals	Therapy and Dosimetrist Team Leads^a^	Allows leads to see how many tasks their group have completed in Aria in a given time frame
Department Schedule	All	Provides contact information and service assignment of the day for acute clinical needs
Dosimetry Current Cases	Dosimetry	Provides workload distribution information across dosimetry team
Downtime Management	Lead Therapists, Physicians, Physics	Assists and facilitates patient replan and triage in the event of proton down‐time
IMRT QA	Physics Residents	Provides record keeping infrastructure and document creation for patient‐specific quality assurance
My Preferences	All	Enables the user to customize their view for their specific needs
On Treatment Whiteboard	Therapy, Dosimetry	Displays CarePaths and metadata of patients on treatment
Special Procedure Scheduling	Physics	Coordinates across CarePaths, Outlook calendars, and clinical service rotations to assign physics to special procedures
Spreadsheet	All	Stores tagged patient data in a searchable and exportable format to answer unique clinical questions
SuperUser	Clinical Programming Group^a^	Enables addition of new users, modification of user rights, and data filter management
Whiteboard	Dosimetry, Physics	Displays each patient's CarePath throughout the treatment planning process along with relevant metadata

^a^ Indicates only these user groups have access to these modules.

#### 2.3.1 Customization

**FIGURE 3:**
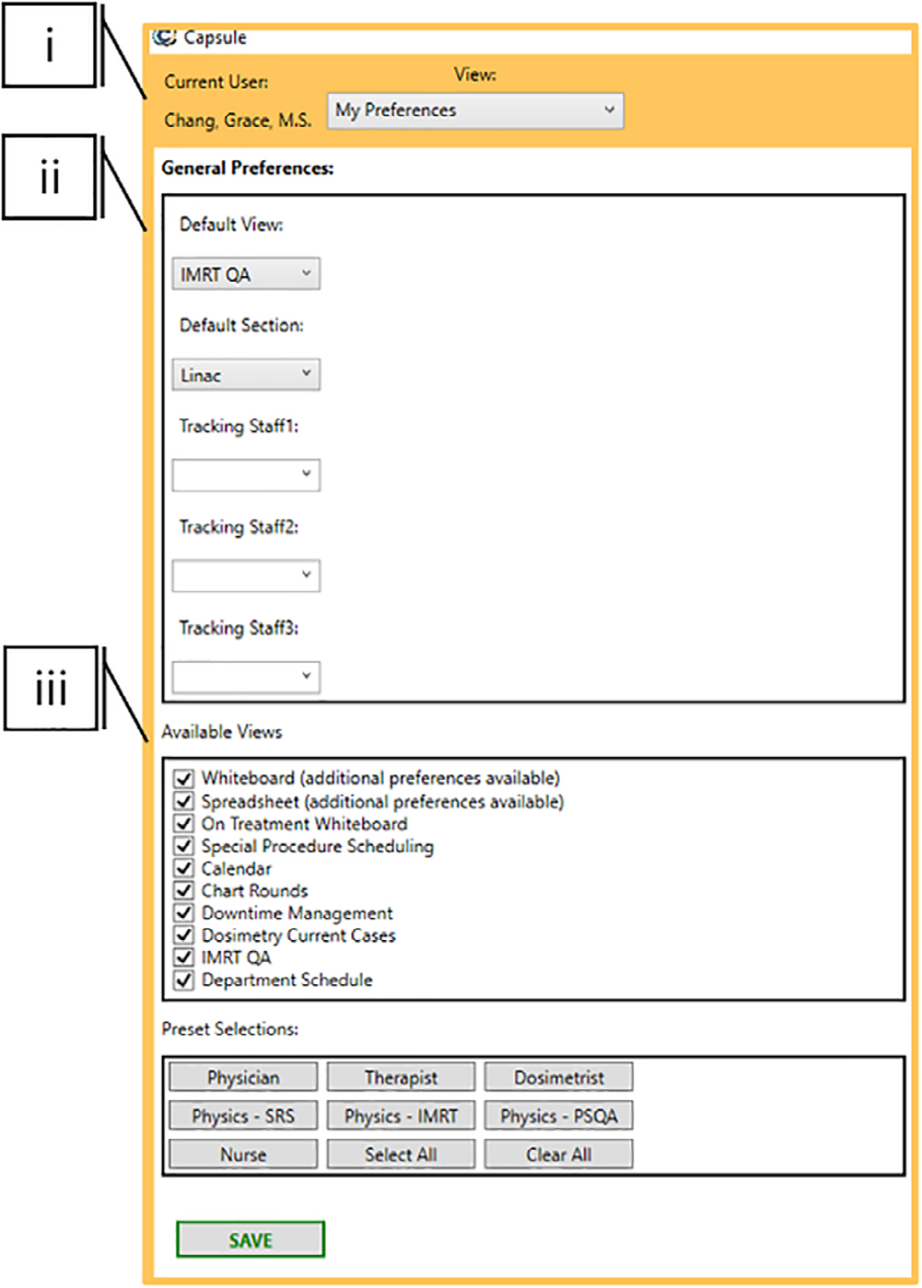
Preference and preset options. Selected customization options. (i) User log‐in information is pulled from the Windows credentials and preserved across workstations. Individuals within the same team in the same clinic may require a range of Capsules functionalities. To accommodate this, presets are designed as a starting point to set preferences most likely relevant to specific roles, including Physician, Therapist, Dosimetrist, and Nurse. (ii) For ease of use, default views are selectable to open first on subsequent access. (iii) Available modules are customizable to reduce cognitive overload. Not displayed are further windows to customize views, including selecting columns available in the Treatment Whiteboard and Spreadsheet modules.

The “My Preferences” Module is the landing page for a new user and prompts them to select default options appropriate to their role (Figure 3). Importantly, the system allows any user to access additional information outside their routine scope by modifying their preferences, thereby promoting cross‐disciplinary awareness and flexibility.

### 2.4 Clinical programming group for in‐house program management

An update policy and team ensure proper quality control. A SharePoint‐based suggestion box is available to all members of the department for new feature requests and bug identification, which are triaged by the Clinical Programming Group (CPG). Three versions of the code support the update workflow: “Testing” is the code that is actively updated by the programmers, “Integration” is for non‐programmer testing and “Production” is for clinical use. The CPG is responsible for development, testing, and routine maintenance. Once an update is released by the CPG, it goes to the “Integration” team, consisting of physicists, dosimetrists, and therapists who conduct validation work. Outcomes are recorded for each item—those marked as FAIL are discussed with the CPG promptly, and fixes are pursued, or implementations are paused. A standardized validation worksheet is stored on a department wide SharePoint for reference.

### 2.5 Workflow efficiency assessment

Workflow efficiency before and after Capsule's implementation was evaluated using quantitative analyses of CarePath task durations and qualitative data from user interviews and surveys. Timestamps for start and end times of all CarePath tasks from “Simulation Finish” to “First Treatment” were queried from the Capsule database (“Plan Preparation”, “Targets”, “Planning”, “Approval”, “Finalization”, and “Physics”). The data was separated into two bins to provide insight into Capsules impact after 1 year of implementation: January 2019 through September 2021, and October 2021 through October 2022. Both mean task time and percentage of tasks completed within 1 day were compared. Statistical outliers in total time from “Simulation Finish” to First “Treatment” were removed using the Median Average Deviation (MAD) outliers method, and significance was assessed using Welch's *t*‐test. A breakdown of treatment plan types is provided.

Supplemental user interviews and surveys were conducted over several weeks. Interview inclusion criteria included: (1) individuals in leadership and Capsule development roles in their teams (therapy, dosimetry, and physics), (2) teams with one or two members, such as physics administrative assistants. While interviews themselves were tailored to the individual, consistent themes were investigated, including perceived ease of tasks with Capsule, the most used module, and how often a module was accessed. A formal survey was then developed for dosimetry teams using Qualtrics (Provo, UT), and a 100‐point slider‐style Likert scale was employed to assess frequency of use. A reduced 5‐point Likert scale was used to identify perceived benefit or detriment of Capsule. Lastly, respondents were asked to list their uses of the Spreadsheet module, and free text was available for additional thoughts on Capsule. Therapists were not surveyed due to limited use revealed in interviews.

## 3 RESULTS

### 3.1 User interviews and surveys

**FIGURE 4:**
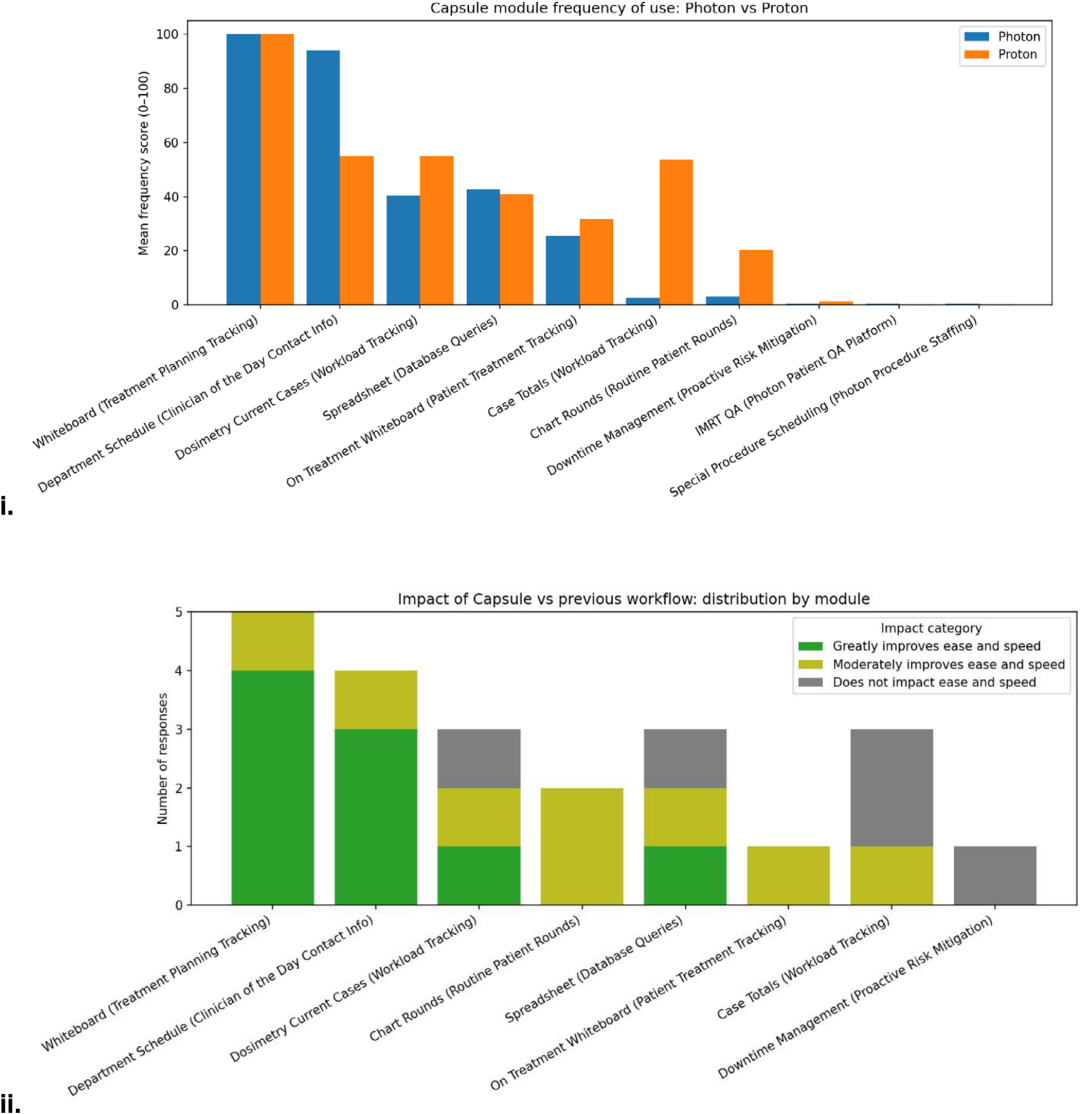
Dosimetry survey results. Likert scale dosimetrist survey results. (i) Of the ten dosimetrists that responded to the survey, the mean frequency of use per module by dosimetry is shown. Frequency of use Likert scale: 100 – Daily or Weekly; 50 – Moderately (once or twice a month); 1 – Rarely: never, once or twice (ii) Six of the ten dosimetrists had worked at our institution prior to Capsules implementation, and only those six were asked but not required to rank the impact of Capsule on the previous workflow. Workflow improvement Likert scale: 5 – Greatly improves ease and speed; 4 – Moderately improves ease and speed; 3 – Does not impact ease and speed; 2 – Slightly hinders ease and speed; 1 – Greatly hinders ease and speed.

A summary of interview and survey results is provided in Figure 4. Ten dosimetrists completed the survey with seven serving the photon clinic and three serving the proton clinic. Sixty percent of respondents worked within their current team at our institution prior to Capsule's implementation. The following summarizes the purposes of selected modules and notable themes that emerged during interviews and surveys.

#### 3.1.1 Department schedule and whiteboard

The Whiteboard and Department Schedule modules were the most frequently mentioned in interviews and the most frequently accessed according to survey results. Respondents reported a frequency‑of‑use score of 100/100 for the Whiteboard module, indicating at least weekly to daily access. Respondents indicated an average of 82.9/100 for frequency‐of‐use of the Department Schedule. In interviews, both dosimetrists and therapists reported using the Department Schedule to identify a contact person for an urgent chart check or physics assistance. In interviews, therapists reported using the standard ARIA CarePath more frequently than Capsule, highlighting that Capsule augments rather than replaces existing CarePaths. In free text response, two dosimetrists expressed a preference for Capsule over a pure whiteboard option also being trialed in our institution, emphasizing that Capsule provides greater utility. One surveyed dosimetrist noted that Capsules Whiteboard substantially reduced manual data entry for the dosimetry team. Reliance on manually updated Excel (Microsoft, Redmond, United States) forms to track patients in the treatment planning stage was removed with the introduction of Capsule.

#### 3.1.2 Down‐time management

To address proton down‐time events, Capsule implemented a standardized triage methodology for facilitating replans (Figure S1).^16^ During a down‐time event, all necessary information for triage is retrieved from the database and displayed on a single screen, including the treatment site, total number of treatments completed and uncompleted, and dates of treatments. Physicians use this data to assign a tier to each patient, which is combined with daily personnel staffing information within Capsule to automatically assign patient replans to photon dosimetrists based on triage level and staff availability. This objective triage tier system allows for efficient down‐time management and has facilitated planning re‐assignments within 1 h during down‐time events in our department. Capsule reduced the screens needed to assign tiers to a lengthy list of patients from one screen per patient to one screen total.

#### 3.1.3 Scheduling and workforce management

The “Special Procedure Scheduling” module covers Total Skin Electron Therapy, Total Body Irradiation, Intraoperative Radiation Therapy, HDR Brachytherapy, Eye Plaques, Stereotactic Radiosurgery Planning, and Spatially Fractionated Radiation Therapy Planning.^17^ To assist physicist coverage scheduling, Capsule searches the relevant data sources (Outlook, ARIA, manually input data) to consolidate information to best identify the next physicist in line and assign them to special procedures. This module reduced the number of screens needed from four to one (Figure S2). Interviews with physics administration revealed Special Procedure Scheduling to be their most frequently accessed module (multiple times per week). Its centralized location also makes it possible for anyone to check who is assigned to which procedure, again reducing the need to send emails or pages. Notably, it provided a system to track how many cases any individual is currently or recently assigned. Workload management is further supported by the Calendar and Dosimetry Current Cases modules. The Calendar automatically displays new start and on‐treatment patient volumes for therapy, whereas Dosimetry Current Cases displays cases per dosimetrist to promote equitable task distribution and workload transparency.

#### 3.1.4 Database

During Capsules development, dosimetrists and physicians requested a module to quickly identify past patients with uncommon treatment scenarios, helping ensure consistent and standardized care even in atypical cases. To streamline non‐routine queries, the Spreadsheet module allows any user to mine the database to generate a patient list based on a range of filters. This augments the traditional ARIA query by including treatment flag data that is visible at a glance. Previously, database queries were funneled to a few individuals with experience performing them in ARIA, and now they can be performed with ease by anyone with minimal training. This provides quick access to specific clinical data trends, aiding executive decision‐making, and resource advocacy. Exported data in .csv files is locked to ensure patient privacy within the hospital's secure servers. Two survey respondents reported using the module to support research activities, including proton stereotactic radiosurgery implementation, standardization, and teaching.

#### 3.1.5 Notification system

Lastly, physician calendars are linked to Capsule along with their contact information. An email is automatically sent to the on‐call physician daily to inform them to check the images for a list of patients for any radiation oncologist who is away from the clinic. Enhancing the notification system is a current focus of the CPG.

### 3.2 Task duration

A total of 8331 individual CarePaths’ start and end times were compiled over the period from 2019 to 2024. When task start times were unavailable, they were taken as the finish time of the preceding task. Tasks that took less than 6 min or more than 2 weeks were omitted and attributed to clerical CarePath errors or anomalous scheduling. Additional outlier removal was done using the Median Absolute Deviation (MAD) method with a modified z‐score threshold of 3.5. MAD is a robust measure of variability that is less sensitive to extreme values than standard deviation, identifying outliers based on their deviation from the median. (Figure S3). Eighteen CarePaths were omitted from before Capsule, and thirty‐seven from 1‐year post‐implementation. One year after Capsule's implementation, total treatment planning duration from “Simulation Finish” to “First Treatment” was reduced by 59.1 h on average (*p* < 0.0001). Most CarePath steps experienced statistically significant reductions in mean task duration with associated Welch's *t*‐test *p*‐values available in Table 2. Pre‐implementation, 86.1% of patients had their “Plan Prep” completed within the first day of “Simulation Finish”. Post‐implementation, the number grew to 95.8%. Target delineation went from 56.4% to 65.1%, being completed within 1 day of “Plan Prep” completion. Finally, the greatest time saving was seen in the “Planning” step, where mean task time reduced by 46.2 h.

**TABLE 2:** CarePath task time before and 1 year after Capsules implementation.

Task	Mean duration before Capsule (h)	Mean duration after Capsule (h)	Change in mean duration (h)	*p*	% of tasks completed within Day 1 before Capsule	%of tasks completed within Day 1 after Capsule
TotalTime	320.9	261.8	−59.1	<0.0001		
Prep	15.1	6.5	−8.6	<0.0001	86.1%	95.8%
Targets	34.8	28.3	−6.4	0.001	56.4%	65.1%
Planning	73.8	27.6	−46.2	<0.0001	24.5%	68.1%
PlanApproval	16.2	12.0	−4.2	0.002	80.1%	87.6%
Finalization	17.9	14.8	−3.1	0.027	82.5%	86.1%
Physics	38.2	37.0	−1.2	0.442	31.9%	48.4%

*Note*: “Before” indicates the time period from January 2019 to September 2021 before the implementation of Capsule. “After” indicates 1 year after the implementation of Capsule, from October 2021 to October 2022 (*n* before = 527, *n* after = 1486). Among the cleaned data set, anatomical site distribution (before vs. after) include Head and Neck (36:138), Brain (77:170), Breast/Chestwall with and without nodes (38:204), Lung (8/121), Pelvis (12:95), Prostate (332:302), Sarcoma (1:11), Abdomen (8:74), Craniospinal irradiation (6:16), Extremity (2:27), Skin (2:32), and Metastases (5:243).

## 4 DISCUSSION

Capsule integrates ARIA CarePaths, Outlook calendars, and scheduling spreadsheets into a unified platform, including a Whiteboard, a Department Schedule, a retrospective database, workload trackers and scheduling assistants. This consolidation, combined with intentional data display, contributed to measurable reductions in treatment‑planning task time, supported by both quantitative analysis and user interviews. Interpretation of task‑duration improvements is limited by concurrent departmental changes, including the introduction of deep learning contour models, additional in‐house automation software, CarePath restructuring, and shifts in patient volume and staffing. To address this limitation, qualitative user interviews provide insight into Capsule's contribution to task facilitation. Pre‐implementation data also relied on Capsules predecessor—a manually updated spreadsheet with lower quality control that was supplemented with ARIA database queries. This data also did not benefit from the treatment flags and anatomical‑site categorizations assigned by treatment planners in the post‑implementation dataset. Ideally, task times would be analyzed by treatment site due to large variations in planning complexity.

Importantly, usability was approached holistically in Capsule's design by emphasizing design principles to enhance the user experience. Thoughtful use of color can guide attention, indicate status or priority, and create visual hierarchies that make navigation more intuitive.^18^, ^19^ For example, consistent color‐coding schemes help users quickly distinguish between task types and urgency levels.^20^, ^21^ Additionally, a clean, well‐organized layout with appropriate contrast and spacing reduces cognitive load, which can allow users to process information more efficiently and make fewer errors. These principles were all incorporated into the iconography and design of Capsule (Figure 2).

Among Capsule's novel contributions are scheduling assistants, workload transparency, and efficient implementation of down‐time triage. These functions are enabled simply by consolidating previously scattered data into a unified database, unlike existing EMRs, which typically require patient‑by‑patient queries and offer limited customization. Capsule's scheduling modules reduced reliance on manual spreadsheets and improved transparency across physics and dosimetry teams using best practices.^6^ By consolidating inputs from the OIS, Outlook, and spreadsheets, tasks that once required multiple systems and communications can now be completed efficiently in one place.

These modules reflect our institution's specific division of tasks and should be re‐evaluated whenever workflows change, or new roles/procedures are introduced. The software architecture, data access methodology and database development presented here are generalizable, whereas the specific flags in the Whiteboard, along with information included in Dosimetry Notes will need to be tailored to an institution's practices. Additionally, the methods of integrating with already active scheduling techniques (in our example, spreadsheets and Outlook) need to be tailored as well.

Capsule exists as a standalone program, but it is implemented within a larger automated ecosystem that supports it. For example, one part of our physics chart check includes ensuring all data is correctly entered into the patients’ Dosimetry Notes. Additional support in the form of standardization in nomenclature during treatment planning, automatic chart checks, and deliberate organization of CarePaths allow Capsule to function smoothly.^22^, ^23^ One potential area for improvement is the expansion of Capsule's notification system. One way it already does this is to send emails to on‐call physicians, as previously mentioned. Additional work is being investigated in developing an urgent case notification tool to ensure all members are prepared for a quick turnaround plan, chart check, or patient‐specific quality assurance.

Although workflow management has not traditionally been a physicist's role, their expertise in quality and systems optimization positions them to lead these innovations. A modern approach to safety and efficiency increasingly calls on physicists to improve not just procedures, but the entire clinical ecosystem.^24^ However, tools like Capsule must address critical limitations in data governance and security. Operating outside the primary OIS can risk noncompliance with patient privacy laws, lack of audit trails, and omission of formal back‐up protocols, raising concerns about data integrity. Capsule mitigates these risks in part by requiring new users to obtain access through a designated Super User, only allowing active ARIA users access, and providing preset customization options based on department roles. Additionally, Capsule does not modify ARIA data; instead, it displays and reorganizes ARIA, Outlook, and manually entered data to facilitate specific workflows. Capsule operates on a hospital server, affording the security system protections and regular data back‐ups employed by the institution. These challenges and risks highlight the need for institutional oversight and robust safeguards when implementing in‐house workflow platforms.

## 5 CONCLUSIONS

The value of a customized data storage and display system, such as Capsule, is two‐fold. First, it has immense potential to enhance workflow efficiency and communication. Routine tasks such as treatment planning, scheduling of special procedures, and identifying the appropriate point of contact for urgent clinical needs are all facilitated by Capsule. Beyond routine clinical tasks, Capsule can support various quality audit and quality improvement projects. Notably, as with any large database, data quality can be an issue. It requires a user to enter things correctly, adequate nomenclature standardization, and the existence of and adherence to standard operating procedure. Capsule relies on standardization tools such as CarePath templates, as well as tools that belong to the larger suite of clinical programs used at our institution that include nomenclature standardization wizards. Additionally, the time needed to update, maintain, and validate the system is substantial as clinical practice evolves. As resources allow, integrating data from Record and Verify systems with scheduling and staffing information enables Capsule to facilitate high‑level communication, comprehensive record‑keeping, and improved departmental efficiency.

## AUTHOR CONTRIBUTIONS

Grace N. Chang organized and conducted user feedback interviews and led manuscript writing. Edward L. Clouser Jr developed the Capsule software and wrote its full codebase. Matthew J. Susen, Pedro R. Lara, and Pamela R. Lemish provided critical user feedback during the development of Capsule. Courtney Buckey designed visuals and user interfaces. Yi Rong, Courtney Buckey, and Quan Chen contributed to its validation, testing, and quality management. Gregory S. Boutis assisted with manuscript writing and provided critical feedback.

## FUNDING

No funding was obtained for this study.

## CONFLICT OF INTEREST STATEMENT

Y.R. is transitioning into the editor‐in‐chief role of JACMP but had no role in the review process for this manuscript.

## ETHICS STATEMENT

No medical data was collected or used in this study.

## Supporting information



Supporting Information

Supporting Information

Supporting Information

Supporting Information

## Data Availability

Data available upon request.
