# Supplementary material for: Four‐year experience with an in‐house treatment management platform to streamline departmental operations in radiation oncology
Source: J Appl Clin Med Phys. 2026 Feb 24;27(3):e70515. doi: 10.1002/acm2.70515 (PMC12931428; doi:10.1002/acm2.70515)
Supplement: Supplementary file 1 — Supporting Information [file ACM2-27-e70515-s003.docx]

**Fig S1. Capsule’s Role in Down Time Management**

**
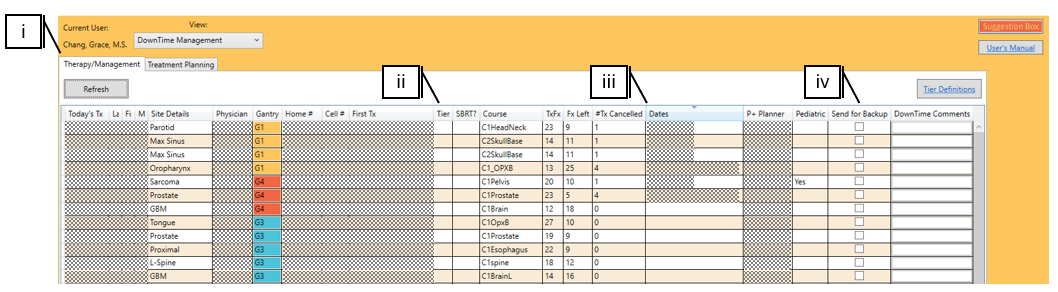
**

**Fig S1.** Example of data within Capsule used during patient triage. (i) Triage starts with physicians assigning a tier in the Therapy/Management tab. (ii) Tier assignment populates once assigned. (iii) Capsule pulls information relevant to the triage tier classification in a consolidated display to aid physician ranking, such as missed treatments, pediatric or adult, dates of treatments, treatment type, and more. (iv) Once assigned a tier and sorted, the patients can be sent to the Treatment Planning stage, where the backup plan can be efficiently assigned to a dosimetrist. This process can be done in an hour, with back up treatment planners assigned and ready to plan the highest priority patients at the one-hour mark.
