# Supplementary material for: Four‐year experience with an in‐house treatment management platform to streamline departmental operations in radiation oncology
Source: J Appl Clin Med Phys. 2026 Feb 24;27(3):e70515. doi: 10.1002/acm2.70515 (PMC12931428; doi:10.1002/acm2.70515)
Supplement: Supplementary file 2 — Supporting Information [file ACM2-27-e70515-s002.docx]

**Fig S2. Special Procedure Scheduling: Brachytherapy**


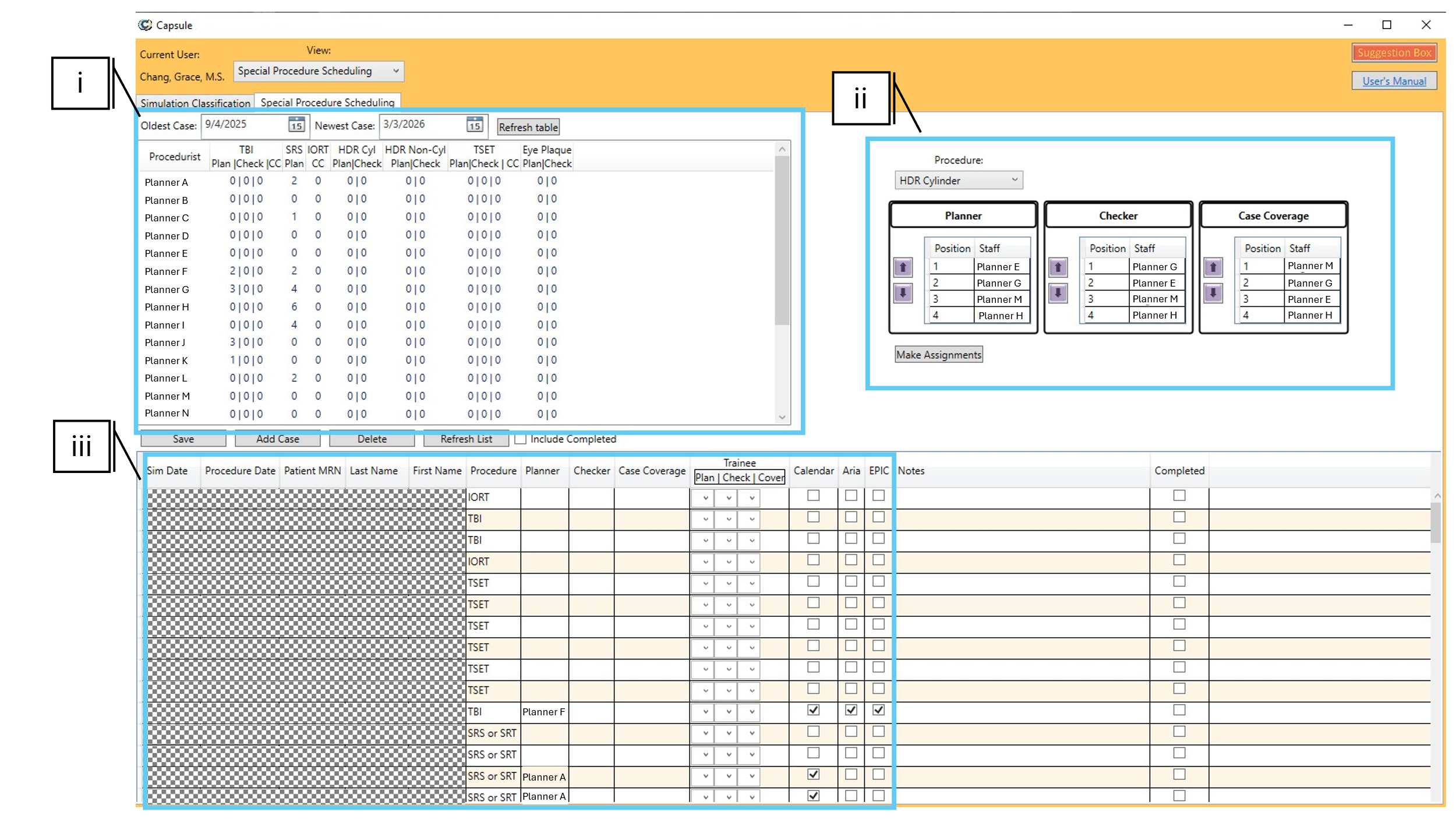


**Fig S2.** Special Procedure Scheduling Tab for an HDR Cylinder. The scheduling workflow is triggered by Capsule automations in most cases. In the simulation classification tab, physics admin assigns a classification (stereotactic radiosurgery (SRS), total body irradiation (TBI), total skin electron therapy (TSET), high dose rate brachytherapy (HDR), intraoperative radiation therapy (IORT) etc.). Then, the Special Procedure Scheduling tab is used to make physics assignments. Each procedure has slightly different workflows, but all leverage Capsules personnel assignment tool. (i) Case tracking at-a-glance is visible for all individuals who are on any given service in the defined date range. (ii) Next in line for coverage is displayed for the various tasks, and only individuals eligible to perform the task indicated by the drop down are included in the rotation (iii) An overview of all special procedure simulations with assigned coverage is available for anyone to review who is covering which patient. The checklist for additional tasks needed to complete scheduling is available as well.
