# Supplementary material for: Four‐year experience with an in‐house treatment management platform to streamline departmental operations in radiation oncology
Source: J Appl Clin Med Phys. 2026 Feb 24;27(3):e70515. doi: 10.1002/acm2.70515 (PMC12931428; doi:10.1002/acm2.70515)
Supplement: Supplementary file 3 — Supporting Information [file ACM2-27-e70515-s001.docx]

**Fig S3. Data Cleaning and Outlier Removal**

**i.
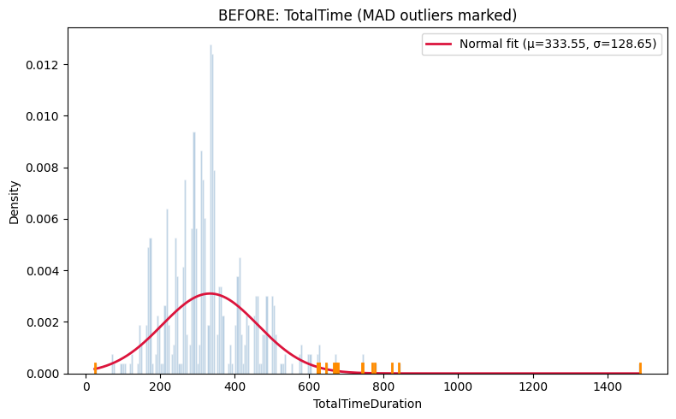
ii.
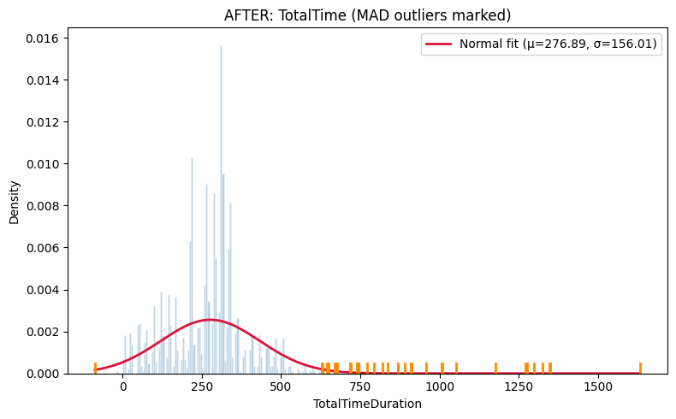
**

**iii.
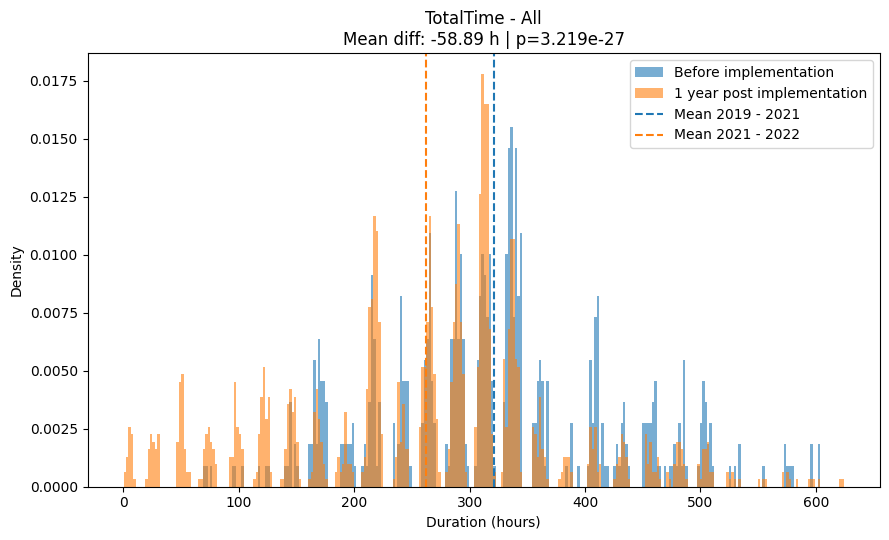
**

**Fig S3.** Distribution of total CarePath time from end of simulation to the first treatment (i) Jan 2019-Sep 2021 and (ii) October 2021-October 2022. Outliers are identified in orange (i. n = 18, ii. n = 37). (iii) The final distributions of total CarePath time are available, with the p-value from Welch’s t-test.
