# Supplementary material for: Four‐year experience with an in‐house treatment management platform to streamline departmental operations in radiation oncology
Source: J Appl Clin Med Phys. 2026 Feb 24;27(3):e70515. doi: 10.1002/acm2.70515 (PMC12931428; doi:10.1002/acm2.70515)
Supplement: Supplementary file 4 — Supporting Information [file ACM2-27-e70515-s004.docx]

**Table S1**. Summary of all data sources referenced by Capsule, and the modules they are leveraged in.

| Source | Data | Module |
| --- | --- | --- |
| Record and Verify | CarePath: task status, individual assigned, treatment progress  Appointments: simulation appointment information | Whiteboard, Chart Rounds, Current Cases, IMRT QA, Calendar, Spreadsheet, Downtime Management, Special Procedure Scheduling |
|  | Diagnosis code | Spreadsheet |
|  | Treatment plan data: name, energy, anatomy, imaging types (4DCT, breath hold), etc. | Whiteboard, IMRT QA, Spreadsheet, Downtime Management, Chart Rounds |
|  | Previous radiation therapy | Spreadsheet, Downtime Management, Chart Rounds, Whiteboard |
| Outlook & Scheduling Spreadsheets | Daily clinical duty assignment | Whiteboard, Department Schedule |
|  | On-site vs Off-site | Department Schedule |
|  | Pager numbers | Department Schedule |
|  | Case coverage | Whiteboard |
|  | PTO | Department Schedule |
| User Input | Treatment type tags: bolus, SRS/SRT, ReTx, breath hold, gated, clinical protocol, insurance status etc. | Whiteboard, Spreadsheet |
|  | Other metadata: number and type of image registrations, previous treatment (outside records) | Whiteboard, Spreadsheet |
|  | Patient specific quality assurance pass rates | IMRT QA |
